# Laser-assisted fabrication of single-layer flexible touch sensor

**DOI:** 10.1038/srep34629

**Published:** 2016-10-05

**Authors:** Seokwoo Son, Jong Eun Park, Joohyung Lee, Minyang Yang, Bongchul Kang

**Affiliations:** 1 Department of Mechanical Engineering, Korea Advanced Institute of Science and Technology, Daejeon 34141, Republic of Korea; 2 Department of Mechanical System and Design Engineering, Seoul National University of Science and Technology, Seoul 01811, Republic of Korea; 3 Department of Mechanical System Engineering, Kumoh National Institute of Technology, Gumi 39177, Republic of Korea

## Abstract

Single-layer flexible touch sensor that is designed for the indium-tin-oxide (ITO)-free, bendable, durable, multi-sensible, and single layer transparent touch sensor was developed via a low-cost and one-step laser-induced fabrication technology. To this end, an entirely novel approach involving material, device structure, and even fabrication method was adopted. Conventional metal oxides based multilayer touch structure was substituted by the single layer structure composed of integrated silver wire networks of sensors and bezel interconnections. This structure is concurrently fabricated on a glass substitutive plastic film via the laser-induced fabrication method using the low-cost organometallic/nanoparticle hybrid complex. In addition, this study addresses practical solutions to heterochromia and interference problem with a color display unit. As a result, a practical touch sensor is successfully demonstrated through resolving the heterochromia and interference problems with color display unit. This study could provide the breakthrough for early realization of wearable device.

Owing to the progress and popularization of portable devices, such as smart phones and tablet computers, the demand of touch sensors for user interfaces is dramatically increasing. From the early pressure-electric type, the touch sensor mechanisms have recently evolved to an electrostatic-capacitive type, which has many advantages such as coincident multiple sensing, high response rate, good durability, and high sensitivity, compared with other competitive methods^1^. A sensor array layer to detect a touch event and a bezel circuit to transmit the electric signal from the sensor to a drive integrated chip (IC) are the key components that directly affect the resulting engineering performance and physical properties such as transparency, resistance, size, and thickness. Indium tin oxide (ITO) is mainly used as a transparent conductive material in sensors, as it is characterized by high transparency within the visible wavelength range^2^,^3^. However, its application is limited to flexible, low-cost, and large area touch sensor because of its brittleness, high material cost, and relatively low conductivity^4^. In addition, the ITO-based device has some disadvantages in the fabrication process because its deposition requires expensive vacuum deposition methods, such as sputtering. Moreover, costly and complicated post-patterning processes, such as photolithography and chemical etching, are necessary. These processes should be repeated to fabricate the bezel circuit.

Most previous studies attempting to resolve the issues of current touch sensors mainly focused on pioneering alternative transparent materials^5^, which can be representatively classified into four categories: conducting polymers^6^, carbon nanomaterials^7^,^8^,^9^, metallic one dimensional nanomaterials^10^,^11^,^12^,^13^, and hybrid materials^5^,^14^,^15^. These attempts do not come up with a definite answer because of the fundamental limitations represented by the limited conductivity and transparency of conducting polymers, the high junction resistance and weak adhesion of carbon nanotubes (CNT), challenging processibility of graphene, excessive haziness and low clarity of metallic nanowires, and process complexity of hybrid structures. Fundamentally, since these approaches have a conflicting relationship between conductivity and transparency, also depending on material thickness, it is considered that concurrently improving them is almost impossible^16^,^17^.

Metal-meshed transparent conductors, another approach adopted when using metals, consist of two dimensionally tangled metal traces of invisibly narrow width. This method provides outstanding advantages in device performance, structural simplicity, and manufacturing availability^16^,^17^,^18^. First, outstanding transparency and clarity are achievable without sacrificing its conductivity, since the transmittance is almost independent from a variation of structure thickness^17^. This approach supports a high design flexibility of touch sensors. Second, an extraordinarily superior mechanical durability is easily achievable due to its own high ductility and elasticity. Lastly, the material cost is lower than other transparent conductors. However, critical problems hinder its cost-effective application. For example, large area metal tracks with a width of less than 5 μm can be almost invisible, but they can hardly be obtained without the use of expensive photolithography processes^16^,^18^,^19^. Although alternative printing methods such as inkjet and roll printings can be used, the minimum resolution that they can provide is larger than that required for such a touch sensor^16^,^20^,^21^. Since the laser sintering process using metal nanoparticles (NPs) fabricates fine metal tracks of less than 10 μm, this method have been also applied to realize the metal mesh-based touch sensor fabrication in spite of a resistive type of touch sensor^22^,^23^. However, this approach is not cost-effective to be applied to practical production. The metal NPs with diameter of less than 5 nm, which are required for sintering efficiency, are more expensive than bulk metals. Moreover, the un-sintered NP solution should be removed and wasted due to the process characteristics. Of course, the material cost can be saved by using larger nanoparticles of more than 5 nm, but it is not appropriate for fabrication of silver structure of a high quality and conductivity because of the less densified structure induced by poor aggregation of the nanoparticles. Visible wavelength lasers used to induce the surface plasmon resonance are also expensive to configure within the laser sintering system in parallel for practical production. Therefore, the laser sintering method has to be replaced with a more efficient solution.

In terms of device structure, the projected capacitive touch sensor of mutual type, which is a representative of the state-of-art, typically requires two perpendicularly crossed sheets of transparent conductive films as vertical and lateral axes, internal layers for electrical insulation between the sensor layers, an outside tempered glass for mechanical protection, and adhesive layers^24^,^25^. This complicated structure results in an increase of device thickness. Therefore, the overall transmittance is degraded because of the accumulative effect of the layer-by-layer light absorption. In addition, as the bezel area, which is usually covered with opaque materials such as carbon black, is needed to place circuits to transmit the electric signal, the resulting available display area is further reduced^26^,^27^. In addition, such a sensor type fundamentally requires an ITO layer for field detection of capacitance, solution processes such as printing cannot be applied to fabricate it and the device is not flexible due to the brittleness of ITO^28^,^29^. Considering the limits of the current state of touch sensors, a breakthrough is highly needed for current manufacturers, as well as future users. To this end, an entirely different approach, involving a novel material, structure, and manufacturing method, is necessary because of their close interconnectivity.

In this paper, to resolve these fundamental and interrelated technical problems, we present single-layer flexible touch sensor incorporating display window, touch sensor, bezel, and other additional layers. It is designed for the realization of an indium-tin-oxide (ITO)-free, bendable, durable, multi-sensible, and thin single layer touch sensor in consideration of the market demands. This single layer structure also enables to save energy by minimizing the optical loss in internal sheets, reducing the weight and improving the productivity. The sensor structure consists of the integrated metal wire networks of self-capacitive sensors and interconnections on a glass substitutive film, as shown in Fig. 1(a). This device concept was realized by near infrared (NIR) laser-induced direct microfabrication based on thermochemical direct metallization of optically enhanced organometallic/nanoparticle hybrid complex, as shown in Fig. 1(b,c). This fabrication method has great merits, such as the huge reduction of material and laser cost compared to conventional laser sintering methods^22^,^23^ via easy in-process generation of ultrafine nanoparticles of 2~3 nm from a low-cost solid-free organometallic solution, process optimized to a NIR laser, one-step production of high-fidelity microstructure, and green manufacturing by low-temperature processing in air. Thus, this study can provide a practical solution to the existing problems characterizing transparent conductive materials and their related applications.

## Results and Discussion

Even if a flexible film could be used as a single substrate which could substitute the window glass of the conventional touch screen panel due to its outstanding mechanical and optical properties, it still has limited direct applications for touch devices because of its weakness for both heat and corrosive chemicals, necessary for the conventional photolithography process^30^. Therefore, the laser-induced fabrication, which enables to fabricate a metallic layer at low temperatures without any corrosive chemicals, is appropriate for that film.

**Figure 1:**
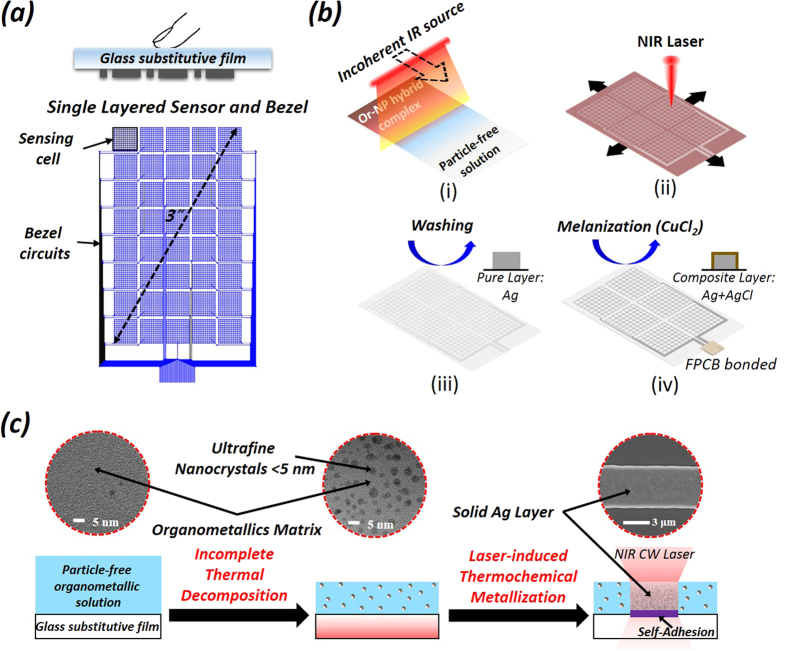
(**a**) Cross-sectional and planar structure of designed touch device. (**b**) Overall fabrication steps using the laser process; (i) *In-situ* generation of nanosalt-pond: uniform nucleation of ultra-fine photonic catalysts by moving linear heater, (ii) Laser processing: selective laser irradiation using focused laser beam and 2D precision translation stage, (iii) Rinse: washing out the unexposed solution using cleaning agent, (iv) Melanization: chemical treatment of silver surface for antiglare and anti-heterochromia problems. (**c**) Schematics of laser-induced microfabrication using thermochemical direct metallization of optically enhanced organometallic/nanoparticle hybrid complex *in-situ* generated from particle-free organometallic solution. The inset is the TEM image of hybrid complex (left) and SEM image of laser processed layer (right).

**Figure 2:**
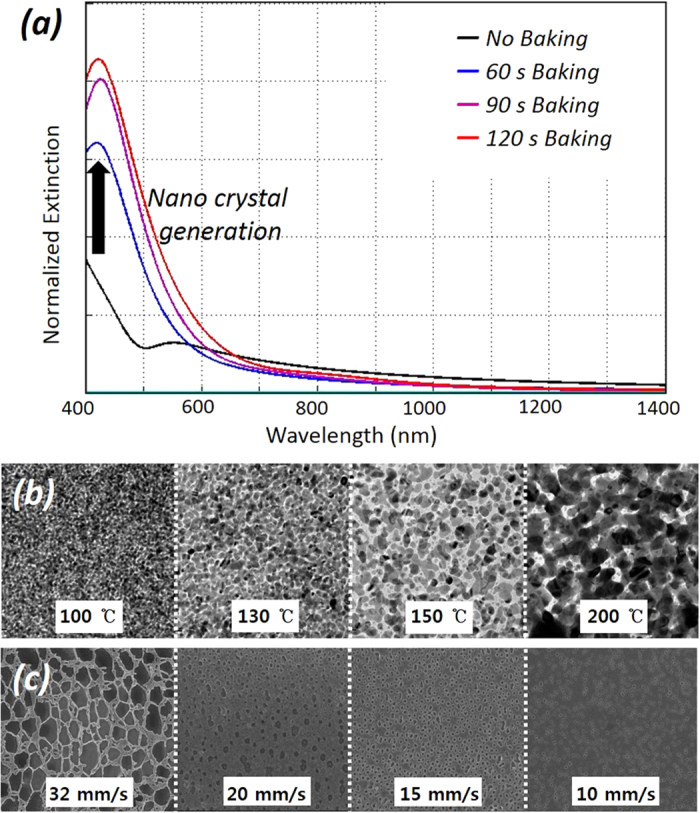
(**a**) Spectral variation of optical extinction of particle-free organometallic solution with prebaking time. (**b**) Transmission electron microscope of particle-free organometallic solution with temperature variation. (**c**) Scanning electron microscope image of the surface of fabricated micro structure with changing a scan rate during laser irradiation.

Unlike those of conventional metal nanoparticle solutions requiring expensive chemical synthesis processes^31^,^32^, the solution used in this study does not have any suspended metal content. In other words, silver, which is ionically bonded (chelate bond) to the carbamate-amine complex (2(R-NHCOO^−^Ag^+^NH_3_^+^-R) + O_2_), is homogeneously dissolved in the organic solution^33^. To raise its laser absorption efficiency, the solutes, which are able to directly absorb light energy and generate heat, are needed as an intermediate phase for energy interaction. In addition to the partial extraction rate of the intermediate nanocrystals from the original solution, the size of the extracted particles can also be controlled by the degree of incomplete thermal decomposition of the organometallic solution, as shown in the inset of Fig. 1(c). Because of this effect, the transparent specimen acquired a semitransparent brown color due to the uniform generation of small nanoparticles. This fact supported by Fig. 2(a), shows that the optical extinction gradually increases with increasing the baking temperature. According to Fig. 2(a), a laser of visible wavelength should be used to sinter the Ag nanoparticles, because the absorption peak is located in the visible wavelength. However, generating the strong absorption on the hybrid complex used in this method poses some critical problems such as the deterioration of surface quality, the limit of pattern thickness, and the increase of photon cost (Supplementary information). Since the mild laser absorption uniformly generates the thermal energy along the thickness direction, prevents the explosive evaporation of organics, and saves the cost of a laser source, the NIR laser is more effective light source for sintering the hybrid complex. The ultrafine Ag NPs of 2~3 nm generated from slight thermal decomposition can improve the absorption of a NIR laser compared to using an original particle-free organometallic solution. Therefore, the ultrafine NPs work as a laser absorber and generate the heat to trigger the decomposition of surrounding organometallics. To investigate this effect crystallographically, the particle nucleation and growth processes were observed by transmission electron microscopy (TEM) in real-time heating. Figure 2(b) shows the *in-situ* transient behavior of the crystallization from an organometallic solution as the chamber temperature increases. At the initial stage of 100 °C, ultrafine single crystal nanoparticles with an approximate size smaller than 5 nm were uniformly generated through nucleation by thermal decomposition. Upon temperature increase, the particles began to grow through thermal agglomeration. In the end, this process led to obtain a fully-grown polycrystalline bulk structure of approximately several hundred nanometers in diameter. As this sequential phase transformation process—involving the pure solid-free solution, organic evaporation, nucleation, growth and agglomeration, and finally polycrystallization—surely requires sufficient time, the laser processing rate is necessarily limited. As the scanning electron microscopy (SEM) images shown in Fig. 2(c), the higher scan speeds of laser spot generated larger holes distributed over the whole surface. Remarkably, residual organics enclosing the particles were present at the highest rate. However, by decreasing the laser scan rate, the size of the holes gradually decreased until they almost disappeared from the surface, creating a denser nanoparticle structure. This can be attributed to the low scan speed, which guarantees sufficient time for both the phase change and organic evaporation. This result shows that a silver structure as smooth and densified as that fabricated by vacuum deposition can be created using this simple ambient process, once the laser-induced direct microfabrication process using organometallic solution is properly controlled^34^. The approach using this nanocrystal-embedded hybrid organometallic complex has the advantage of a great reduction of material cost compared to using a conventional nanoparticle solution, because it can easily make ultrafine photonic catalysts to interact with an incident laser without any efforts to synthesize ultrafine nanoparticles which become chemically unstable^34^. This can be compared to an effect to gathering salt powders in salt-pond instead of using a particles suspended in a solution.

Notably, the material absorption properties can be modified during real-time process. Typically, the absorption rate of a material at a specific wavelength is regarded as an original characteristic of the material. Thus, considerable effort, time, and cost are required to find the optimal laser-processing conditions for a specific material, even when the nano-colloidal solution is used, as the synthesis method needs to be modified to change the overall absorption rate^35^,^36^. However, as shown in Fig. 2(a), this method can easily and broadly control the absorption rate over a wide spectral range, from ultraviolet to near-infrared, by just changing the prebaking time. Hence, the optimization to find the critical interaction point is quite simple. As a result, a heat treatment for 90 s at 100 °C was determined as the optimal pre-baking condition.

**Figure 3:**
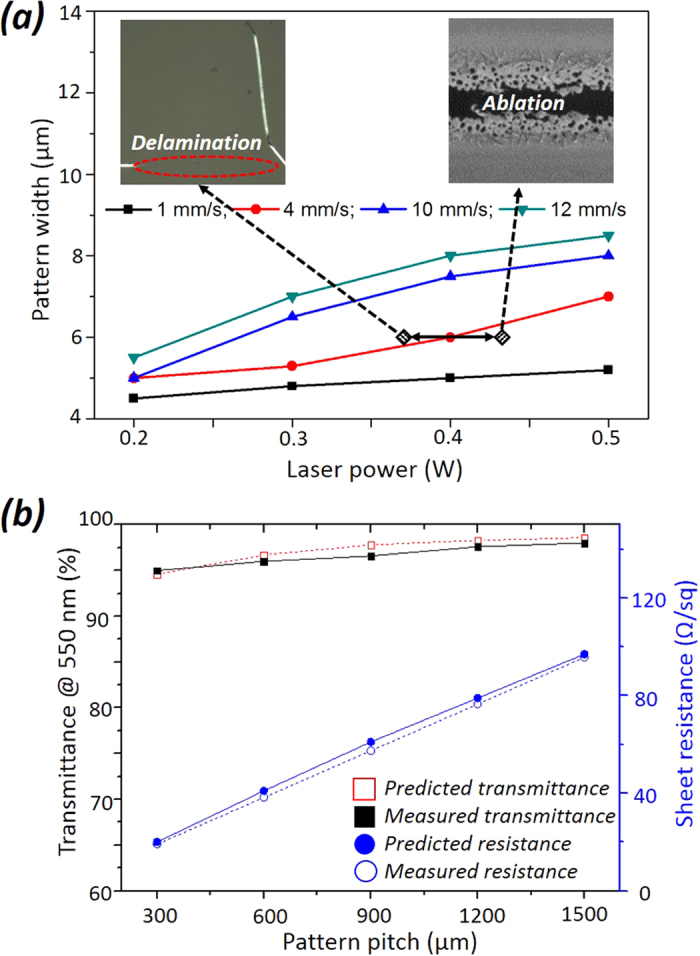
(**a**) Characteristics of laser direct pattering of particle-free organometallic solution. Inset shows the ill-conditioned patterns. (**b**) Variation of transmittance and sheet resistance of silver wire network sensor with respect to pattern density.

Figure 3(a) shows the variation of pattern width with respect to both the laser power and scan rate. In general, this result seems to closely follow the thermal energy relationship that characterizes typical laser processing^37^. When the relationship was not followed, the processed structure was either peeled off from the substrate, owing to incomplete solidification, or partially ablated, owing to the excessive increase of the energy input, as shown in the insets of Fig. 3(a). In addition, since the pattern width sensitively changed with the laser power fluctuation at fast scan rates above 4 mm/s and lower laser speeds, a narrower width was achievable, and the laser scanning experiment were performed by only changing the laser power while maintaining the scan rate at 1 mm/s (Fig. 3(a)). Under this condition, the specific resistance of 5.8 μΩ∙cm was recorded, which is close to that of the bulk and comparable to that of other materials fabricated with alternative manufacturing methods. Based on the basic characteristics of this method, the line width of the silver conductor for the sensors was 5 μm. Otherwise, that of the bezel was 10 μm as the purpose of this step was to prevent disconnections between the sensor node and bezel interconnections and to prevent electrical loss while delivering the electrical signal to the IC chip.

The transmittance and sheet resistance of this sensor structure are easily predicted using simple geometrical relationships^38^,^39^. The transmittance is calculated by the fraction of subtracting the area shaded by the metal line, i.e., the fill factor, from the area of the sensing part. The sheet resistance is proportional to the specific resistance but inversely proportional to the thickness and area of the metal lines. According to this relationship, the designed optical and electrical properties with respect to the pitch width representing the fill factor are depicted in Fig. 3(b). To perform a comparison with the actual properties of the fabricated sensor, both the transmittance at a specific wavelength (550 nm) and the sheet resistance were measured, and are also included in Fig. 3(b). The designed values are in good agreement with the experimental results, and the transmittance and sheet resistance exhibited a linear relationship with respect to the pitch width. According to these results, a pitch of 300 μm was found to be the most appropriate for the subpixel mesh, as the sheet resistance was minimized without excessively reducing the transmittance. Even though a change in thickness can modify these characteristics, the thickness was fixed to 200 nm, considering both the ability to uniformly deposit the solution and the limitation of the laser penetration depth.

In contrast to conventional capacitive touch sensors that have multi-layer structures, a single layer structure was proposed in this study. As the multilayer configuration requires two patterning steps, entailing a vertical axis and a lateral axis, and the insertion of insulator layers between the sensor layers, the process is highly complicated. The sensor structure of the proposed self-capacitive type was motivated by simple manufacturing and slimness of device. This device concept is definitely different from conventional approaches as the individual sensing pixel directly detects a variation of capacitance and its size determines the sensing resolution. Such a single layer structure has a great advantage in terms of fabrication; for example, only a single patterning process is required to fabricate both the sensors and bezels without changing the processing area.

**Figure 4:**
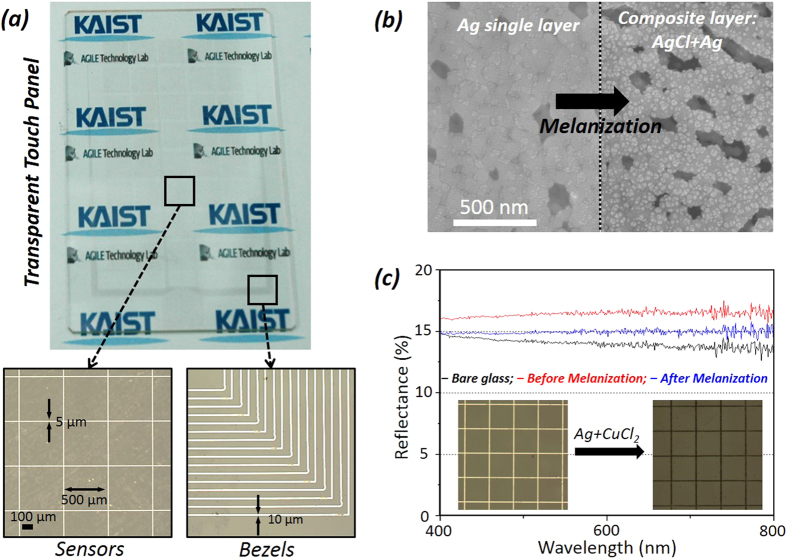
(**a**) Transparent touch sensor panel on a copy paper and partially magnified microscope images (left: sensing pixel and right: bezels). (**b**) SEM and microscope image of silver structure before and after melanization treatment for antiglare and invisibility. (**c**) Spectral reflectance behavior on melanization treatment. Inset is the microscopic image of corresponding silver structure.

The individual pixels are composed of a group connection of arrays of square sub-pixel, which are shaped by a closed loop metal wire mesh with a size of 300 μm. The net size of a pixel is a square of 7 × 7 mm^2^. The subpixel density can be changed by controlling the line pitch, so that the transmittance and the resistance are respectively tuned. Figure 4(a) shows the completed touch sensor including 40 sensing pixels and bezels. Each sensing pixel was connected to the corresponding bezel line. The tails of the bezels bonded to the flexible printed circuit board (FPCB) are laterally arranged with a space of 240 μm on the bottom region of the substrate, as shown in the inset of Fig. 4(a). The drive integrated circuit (IC) periodically transmits the electric wave signal to all sensing cells and monitors the variation of its frequency on real time, because the frequency is changed by capacitance variation on cell. The measured value for each electrode is compared to the baseline against a defined touch threshold value (Supplementary information).

The surface roughness, another important parameter to evaluate the device quality, was found in the range of ~5–6 nm. However, this outstanding morphological quality negatively affects the application as the transparent conductor for touch sensors, as it become more distinguished and glared compared to a background color image owing to the specular reflection of light on the mirror-like smooth metal surface at a specific angle. This may disturb the clear viewing when users look at the display image through the sensor. To minimize this negative effect, the surface should be rougher and induce diffuse reflection. In addition, the metallic color should be changed to black, as it shows the chromatic heterogeneity with that of the color display unit. To address these practical requirements, a slight change of the chemical composition of the surface of the Ag mesh structure was introduced by employing an aqueous solution of copper chloride (CuCl_2_)^40^,^41^. By immersing the laser-processed specimen in the solution, only the silver ions on the outer surface combined with the chloride ions, producing silver chloride (AgCl). As a result, the surface became rougher and appeared darker, as shown in Fig. 4(b,c). Consequently, the resultant light reflectance on the surface decreased by approximately 4% over the whole visible wavelength range, and the visibility of meshes also notably decreased.

**Figure 5:**
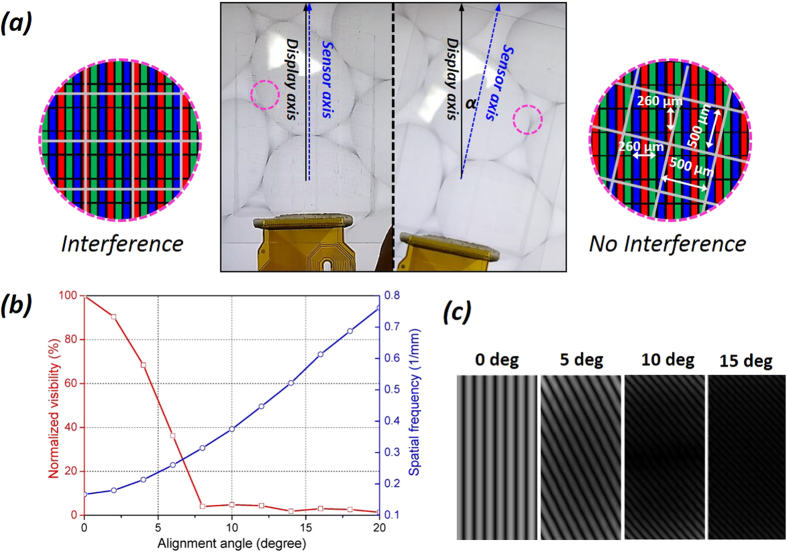
(**a**) Moiré reduction effect by orientation of silver wire networks with respect to display pixel arrays; left: coincident (α = 0°) and right: misaligned (α~11°). Inset is the schematics on interference occurrence between the wire networks (gray) and red/green/blue color pixels. (**b**) Simulation result of spatial frequency and visibility of moiré pattern with respect to alignment angles and (**c**) corresponding image of moiré fringes.

Another practical issue arises when the sensor is integrated with the display pixels at a specific angle, as shown in Fig. 5(a). In this case, a moiré pattern distorting the background image is also generated by the light interference between sensing pixels and display pixels^42^. In principle, moiré patterns are inevitable in display systems with metal-meshed touch sensor, but their frequency and intensity can be minimized by properly tuning the periodical geometries of each unit. The typical method to reduce this unwanted effect is to precisely orient each component layer of multilayer touch sensor with respect to the display pixel arrays, respectively. However, in the current method, the moiré problem can be simply resolved by just once rotating the integrated sensor panel for the display unit. Figure 5(b) shows the variation of visibility and spatial frequency of moiré pattern with respect to alignment angles between display unit and touch sensor, which is calculated based on the geometry of color pixels and silver mesh electrodes (Fig. 5(a))^43^. As the alignment angle increases from 0 to 20°, the visibility drops dramatically then it is saturated from an angle of 8° and the spatial frequency gradually increases. Because of the large decline of the visibility and the continuous decrease of spatial period, the moiré patterns become visually undistinguishable then disappears in the large alignment angles, as shown in Fig. 5(c). The minimum angle undetectable range is placed above an angle of 10°, which almost corresponds with the result finding from the experiments (misalignment angle: 11°).

**Figure 6:**
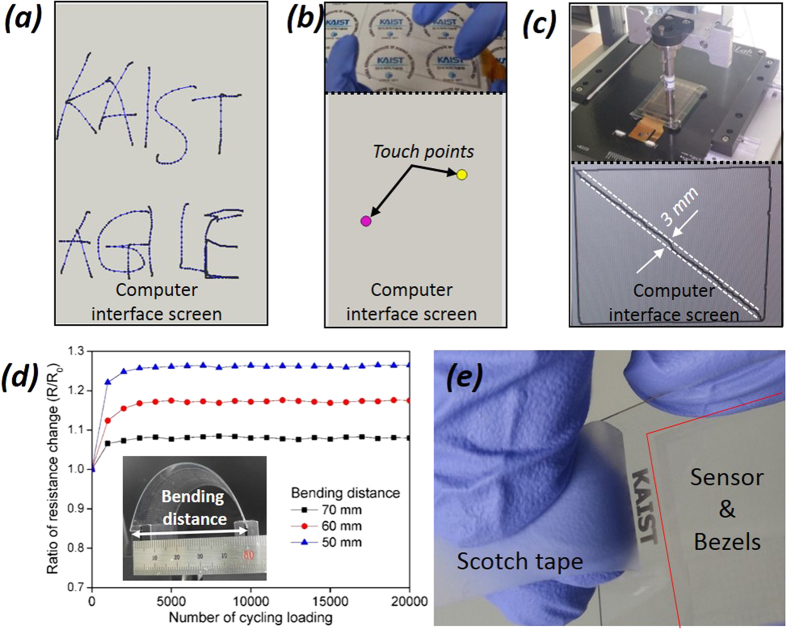
Touch demonstrations and evaluation. (**a**) Drawing of arbitrary letters (KAIST AGILE), (**b**) Multiple detecting of the touch sensor, (**c**) Test of detection accuracy and directionality, (**d**) Cycling bending test for the laser processed touch sensor, as a function of bending distance. The bending rate was 500 mm/min. The inset is a photograph of bending test setup. (**e**) Peeling test using a conventional scotch tape after 100 times.

To evaluate the practical usage of the device, some tests were attempted by writing arbitrary letters, which were well recognized, as shown in Fig. 6(a). In addition, it was verified that this device can simultaneously detect multiple touching, as shown in Fig. 6(b). For a quantitative evaluation of its resolution and linearity, an additional measurement was performed by drawing a rectangle with a diagonal line with respect to the pixel array, using the suitable equipment. As shown in Fig. 6(c), good linearity and a resolution of approximately 3 mm were recorded, in spite of just using a 40 pixel sensor. As a result, a single layer flexible touch sensor module was successfully produced via the one-step laser-induced fabrication method. Unlike the conventional ITO-based touch sensors, which are quite brittle, this device can be circularly bent and easily change its own shape because of the mechanical elasticity of the metal itself. The flexibility and durability of laser processed electrodes were quantitatively evaluated by monitoring the resistance dependence on the bending distance with a cycling rate of 500 mm/min. As show in Fig. 6(d), the electrical conductivity exhibited excellent durability under a bending distance of 50 mm (a curvature of 18 mm) even if it was slightly decreased under bending conditions. Here, the glass substitutive film was bended within a bending distance from 50 mm to 70 mm because the film surface began to crack from the bending distance below 50 mm. The film shows a weak flexibility, but it does not matter because the bendability can be further improved by decreasing the film thickness. In addition, the electrodes showed an outstanding adhesion property on the film, as show in Fig. 6(e). This good reliability results from the formation of a fully dense silver structure and the laser-induced instantaneously thermal merging between the electrodes and the film. Most importantly, the function of touch sensing is well retained even when the device is bent, as shown in Supplementary movie. Consequently, this fabrication technique can be applied to typical tempered glass and polymer films without the change of the fabrication method.

## Conclusion

The approach described in this paper, which involves an alternative material, newly designed structure, and innovative fabrication method, can have a major impact on the current display and related devices industry. In this work, single-layer flexible touch sensors characterized by ITO/photolithography-free, single layer, bendable, durable and multi-touchable devices were realized by using a cost-effective and one-step laser applied fabrication, which are crucial features for high-yield industrial production. This study provides a solution to a long-running dilemma afflicting the display industry, showing that conventional microfabrication processes can be innovatively improved.

## Methods

A glass substitutive polymer film (REPTY DC 100, Riken Technos Co. Ltd.) with a thickness of 500 μm, which is characterized by a high surface hardness (pencil hardness of 9H, Supplementary information) and optical properties (transmittance: 91% and haze: 0.2%) equivalent to glass, was used as the substrate of single-layer flexible touch sensors^30^. But the process must be performed under a temperature of 150 °C because it begins to deflect above it. Before the main laser process, the substrate was cleaned by using several chemical procedures, such as ultrasonic vibration assisted-immersion in acetone and distilled water for 10 min, and subsequent drying with N_2_ gas. Then, using a commercial spin coater, the substrate was coated with an organometallic solution used as a silver precursor for the laser process and it was supported by Inktec Co. Ltd (CO-011, 10 wt %,). The cost is much cheaper than conventional nanoparticle solutions as an approximately 300 dollars per liter, even if it is depending on the quantity ordered. The spinning rate was controlled with the required coating thickness within the range of ~1000–3000 rpm. Subsequently, the specimen was uniformly heated by using a linear heating source of a maximum power of 100 W. Here, the vertical distance between the light source and the substrate, heating power, and translation rate were precisely controlled, in accordance with the required thermal treatment condition such as a static heating for 90 s at 100 °C. Mouse bite like defect depends on the non-uniformity of the coating thickness and the degree of pre-thermal treatment over the whole processing area. These problems were prevented by designing bezel electrodes wider than the sensor electrode and by using a uniform thermal treatment based on linear scanning of IR radiation, respectively.

An objective lens with a magnitude of 20 (Mitutoyo Co.) was integrated with a continuous wave 1070 nm wavelength fiber laser of 10 W maximum output power (IPG Photonics Gmbh). The laser spot focused on a polished working plate, which could precisely translate in three dimensions. The laser power and the working plate moving rate were automatically controlled to change the required patterned width and drawing path, respectively. The laser process to fabricate a complete touch sensor unit of 3 was divided into two sub-steps; the first step involved the fabrication of sensor arrays, while the second step interconnected the bezel circuits for transmitting signals to a drive IC. Technically, a laser diameter of 5 μm was used for the sensor fabrication by both tightly defining the focusing zone and precisely controlling the laser power to minimize the pattern width. The effective laser diameter for the bezel circuit fabrication was maintained at approximately 10 μm by only slightly increasing the laser power without changing the corresponding optics. After the laser process, the specimen was ultrasonicated in a normal hexane solution for 10~20 s to selectively remove the solution in the laser-unexposed region. The specimen was then immersed in a copper sulfate aqueous solution (H_2_O:CuCl_2_ = 100 g:0.1 g) for 30 s to form silver chloride (AgCl) on the surface.

Anisotropic conductive film (ACF) bonding, which is a kind of soldering process to electrically connect the bezel circuits to the FPCB with drive IC using a conventional flip chip bonder, was used. To prevent short-circuit problems between the bezels during bonding, an ACF containing a solder ball of 10 μm in diameter was employed. The IC and software used for driving the self-capacitive sensor fabricated were an initial engineering sample developed by ATLAB Co.

## Additional Information

**How to cite this article**: Son, S. *et al*. Laser-assisted fabrication of single-layer flexible touch sensor. *Sci. Rep.* **6**, 34629; doi: 10.1038/srep34629 (2016).

## Supplementary Material

Supplementary Information

Supplementary Video
