# Supplementary material for: Laser-assisted fabrication of single-layer flexible touch sensor
Source: Sci Rep. 2016 Oct 5;6:34629. doi: 10.1038/srep34629 (PMC5050410; doi:10.1038/srep34629)
Supplement: Supplementary Information [file srep34629-s1.pdf]

Supplementary information

Laser-assisted fabrication of single-layer flexible touch sensor

Seokwoo Son[†§], *Jong Eun Park*[†], *Joohyung Lee*[††], *Minyang Yang*[†]*, *Bongchul Kang*[†††§*]

[†] Department of Mechanical Engineering, Korea Advanced Institute of Science and Technology, Daejeon 34141, Republic of Korea,

[††] Department of Mechanical System and Design Engineering, Seoul National University of Science and Technology, Seoul 01811, Republic of Korea,

[†††] Department of Mechanical System Engineering, Kumoh National Institute of Technology, Gumi 39177, Republic of Korea.

[§]These authors contributed equally to this work.

*Address correspondence to myyang@kaist.ac.kr (M. Yang); kbc@kumoh.ac.kr (B. Kang)

Movies S1 shows the working demonstration of single-layer flexible touch panel. Available at http://www.nature.com/srep

Role of Ag NPs and its interaction with NIR laser irradiation in laser-induced metallization of hybrid complex.

Ag nanoparticles (NPs) have the absorption peak in visible wavelength range due to surface plasmonic resonance effect. In general, a laser of visible wavelength should be used to sinter the Ag NPs. However, generating the strong absorption on the organometallics/nanoparticle hybrid complex used in this work makes some critical problems such as the deterioration of surface quality, the limit of pattern thickness, and the increase of photon cost. Since most of silver still exist in the form of organometallic solution even if u ultrafine Ag NPs are generated and dispersed sparsely in the solution, the intensive absorption of light induces the explosive evaporation of the un-decomposed organometallic solution and results in the formation of voids. As a result, the surface quality is deteriorated. Next, NIR-lasers deeply penetrate into the Ag NPs film and generate uniform heat due to long laser absorption depth. The extinction coefficient of Ag NPs at NIR region (~1000 nm) is 1~2 order lower than that of surface plasmon resonance (SPR) region (~430 nm), which is supported by figure 2-(a) of the manuscript. The long absorption depth caused by the low value of extinction coefficient enables the transversally extended sintering of Ag NPs, which uniformly generates the thermal energy along the thickness direction. This homogeneously distributed heat is effective to aggregate Ag NPs deep through the bottom. So the structure thickness increases by using the NIR laser compared to using visible wavelength laser for strong absorption. And the photon (laser source) cost is also significant to configure the laser sintering system in parallel for practical mass production. Since, however, the previous works are based on surface plasmon resonance induced by visible wavelength lasers whose cost is 1~2 order higher than NIR wavelength lasers used in our study [22,23], the light source for sintering should be also replaced into a more cost-effective laser for practical application of the laser sintering process.

Comparative pencil test of a conventional polymer and glass substitutive film.

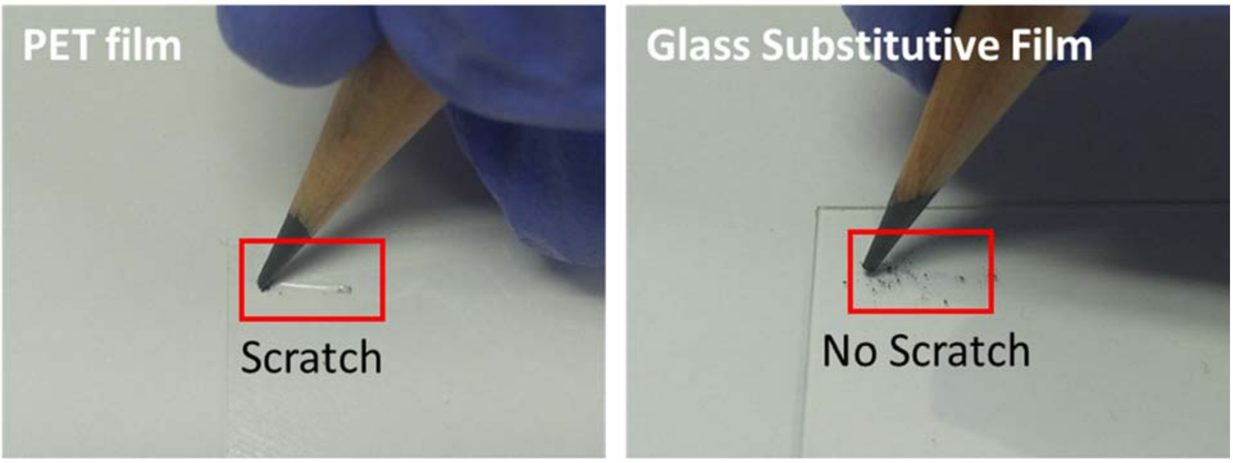

Figure S1. Hardness test using a 9H pencil (left: on PET film; right: on glass substitutive film)

Principle and operation mechanism of one plastic touch sensor

Single-layer touch sensor we presented is mainly composed of four parts, such as planar arrays of capacitive sensing cell, bezel electrodes, a FPCB, and a drive IC. The individual sensing cell which has an independent closed circuit system is connected with a bezel electrode. The sensing cell detects the capacitive load, relative to circuit ground and the signal delivers to the drive IC through bezel electrodes and a FPCB. The capacitance of each X and Y axis electrode on a cell can be independently measured. Measuring the capacitance of each individual sensor electrode provides for the determination of the two dimensional location of a single touch event in progress. The drive IC periodically transmits the electric wave signal to all cells and real time monitors the variation of its frequency because the frequency is changed by immediately responding capacitance variation on cell. The measured value for each electrode is subtracted from a previously acquired no-touch value for the respective electrode and is compared from baseline against a defined touch threshold value. This operation mechanism is expanded to the multi-touch sensing by correlation of multiple X and Y touched electrodes into multiple two dimensional touch coordinates.

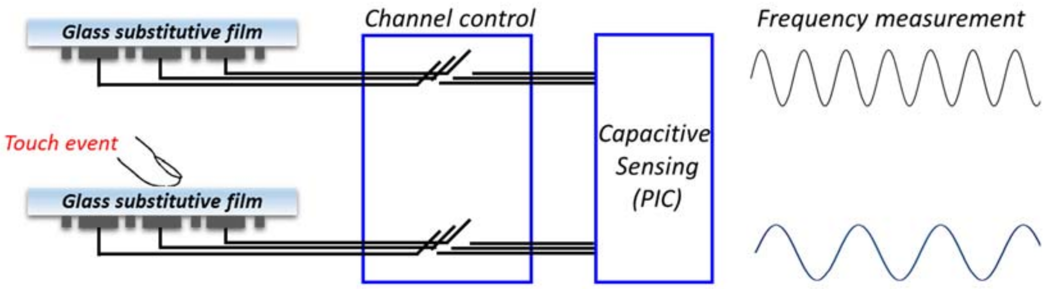

Figure S2. Schematic diagram of interface between touch sensor and drive IC.
